# Identification of novel influenza A virus exposures by an improved high‐throughput multiplex MAGPIX platform and serum adsorption

**DOI:** 10.1111/irv.12695

**Published:** 2019-11-08

**Authors:** Zhu‐Nan Li, Emily Cheng, Eugenie Poirot, Kimberly M. Weber, Paul Carney, Jessie Chang, Feng Liu, F. Liaini Gross, Crystal Holiday, Alicia Fry, James Stevens, Terrence Tumpey, Min Z. Levine

**Affiliations:** ^1^ Influenza Division National Center for Immunization and Respiratory Diseases Centers for Disease Control and Prevention Atlanta GA USA; ^2^ Battelle Memorial Institute Columbus OH USA; ^3^ New York City Department of Health and Mental Hygiene New York NY USA

**Keywords:** hemagglutinin, influenza, MAGPIX, serum adsorption, subtype

## Abstract

**Background:**

The development of serologic assays that can rapidly assess human exposure to novel influenza viruses remains a public health need. Previously, we developed an 11‐plex magnetic fluorescence microsphere immunoassay (MAGPIX) by using globular head domain recombinant hemagglutinins (rHAs) with serum adsorption using two ectodomain rHAs.

**Methods:**

We compared sera collected from two cohorts with novel influenza exposures: animal shelter staff during an A(H7N2) outbreak in New York City in 2016‐2017 (n = 119 single sera) and poultry workers from a live bird market in Bangladesh in 2012‐2014 (n = 29 pairs). Sera were analyzed by microneutralization (MN) assay and a 20‐plex MAGPIX assay with rHAs from 19 influenza strains (11 subtypes) combined with serum adsorption using 8 rHAs from A(H1N1) and A(H3N2) viruses. Antibody responses were analyzed to determine the novel influenza virus exposure.

**Results:**

Among persons with novel influenza virus exposures, the median fluorescence intensity (MFI) against the novel rHA from exposed influenza virus had the highest correlation with MN titers to the same viruses and could be confirmed by removal of cross‐reactivity from seasonal H1/H3 rHAs following serum adsorption. Interestingly, in persons with exposures to novel influenza viruses, age and MFIs against exposed novel HA were negatively correlated, whereas in persons without exposure to novel influenza viruses, age and MFI against novel HAs were positively correlated.

**Conclusions:**

This 20‐plex high‐throughput assay with serum adsorption will be a useful tool to detect novel influenza virus infections during influenza outbreak investigations and surveillance, especially when well‐paired serum samples are not available.

## 1 INTRODUCTION

Influenza viruses constantly undergo antigenic drift and shift leading to the periodic emergence of novel viruses that can cause human infections. Humans also have complex immune history to influenza because of repeated infections and vaccinations to seasonal influenza. Hemagglutination inhibition (HI) and virus microneutralization (MN) assays are gold standards in influenza serologic studies, and both primarily detect antibodies to hemagglutinin (HA) of influenza viruses. Seroconversion with a ≥ 4‐fold rise in HI or MN antibody titers is indicative of recent influenza virus infection or vaccination.^1^, ^2^ In recent years, magnetic multiplex fluorescence microsphere immunoassays (MAGPIX) have been developed to investigate antibody profiles following influenza vaccination/infection and aid in the diagnosis of influenza.^3^, ^4^, ^5^ Previously, we developed a high‐throughput MAGPIX assay with serum adsorption using two ectodomain recombinant HAs (rHAs) from A(H1N1)pdm09 (A/California/7/2009, CA/09) and A/Perth/16/2009 (Perth/09, A(H3N2)) to identify novel influenza exposures when well‐paired human serum samples were available.^4^ However, during novel influenza virus outbreak investigations, paired serum collection may not always be feasible, posing challenges to determine influenza virus infections through serology.

Recently, we identified the second case of A(H7N2) human infection from cats using single serum samples collected from workers/volunteers in New York City animal shelters where A(H7N2) influenza A viruses caused the first documented cat‐to‐human transmission.^6^, ^7^, ^8^ For novel influenza surveillance at the animal‐human interface, we also analyzed paired sera collected from workers in a live poultry market in Bangladesh where multiple avian influenza viruses co‐circulate among poultry and can cause sporadic A(H5N1) influenza virus infections in humans.

In this study, we improved the MAGPIX assay by expanding the multiplex to 20 antigens using 19 globular head domain (GH) HA1 and/or ectodomain (Ecto) rHAs from 9 subtypes of influenza A viruses (H1N1, H2N2, H3N2, H5N1, H7N2, H7N7, H7N9, H9N2, and H13N9), 2 lineages of influenza B viruses (Yamagata and Victoria lineages), and a protein A control (Table 1). Furthermore, given that past exposures to seasonal influenza viruses through infections or vaccinations in the human populations can often cause cross‐reactive antibody responses to novel influenza viruses and complicate the interpretation of serologic data,^4^ we also expanded serum adsorption from two to eight ectodomain rHAs to reduce cross‐reactivity. This multiplex MAGPIX platform with serum adsorption can be a valuable tool to detect novel influenza virus infections during influenza surveillance and outbreak investigations.

**Table 1:** Correlations between MN titer and MFI value against recombinant ectodomain (Ecto) and/or globular head domain HA1 (GH HA1)

#	Code	Ecto/GH HA1	Subtype	Strain	Pearson's *r* value (n = 119)^a^	Pearson's *r* value (n = 58)^b^
1	H1.Mar.43 Ecto	Ecto	A(H1N1)	A/Marton/43	−0.13	0.31
2	H1.Tx.91 Ecto	Ecto	A(H1N1)	A/Texas/36/91	0.02	0.071
3	H1.CA.09 Ecto	Ecto	A(H1N1)	A/California/7/2009	−0.1	0.32
4	H1.CA.09 GH	GH HA1	A(H1N1)	A/California/7/2009	−0.13	0.23
5	H2.Jap.57 GH	GH HA1	A(H2N2)	A/Japan/305/57	−0.13	−0.18
6	H3.HK.68 Ecto	Ecto	A(H3N2)	A/Hong Kong/1/68	−0.1	−0.37
7	H3.LND.86 Ecto	Ecto	A(H3N2)	A/Leningrad/360/86	0.088	−0.26
8	H3.Per.09 Ecto	Ecto	A(H3N2)	A/Perth/16/2009	0.09	−0.41
9	H3.Per.09 GH	GH HA1	A(H3N2)	A/Perth/16/2009	0.1	−0.44
10	H5.VN.04 GH	GH HA1	A(H5N1)	A/Vietnam/1203/2004	−0.08	0.5
11	H5.Ind.05 Ecto	Ecto	A(H5N1)	A/Indonesia/5/2005	−0.09	**0.78** c
12	H5.Ind.05 GH	GH HA1	A(H5N1)	A/Indonesia/05/2005	−0.03	**0.78** c
13	H7.NED.03 GH	GH HA1	A(H7N7)	A/Netherlands/219/2003	0.26	−0.13
14	H7.SH.13 GH	GH HA1	A(H7N9)	A/Shanghai/2/2013	0.32	−0.03
15	H7.NY.16 Ecto	Ecto	A(H7N2)	A/New York/108/2016	**0.63** c	−0.12
16	H9.HK.09 GH	GH HA1	A(H9N2)	A/Hong Kong/33982/2009	0.09	0.02
17	H13.DE.04 GH	GH HA1	A(H13N9)	A/shorebird/DE/68/2004	0.04	−0.2
18	B.Bris.08 GH	GH HA1	B/Victoria	B/Brisbane/60/2008	−0.01	0.07
19	B.Wis.10 GH	GH HA1	B/Yamagata	B/Wisconsin/01/2010	0.01	0.16
20	PA	Ctrl	N/A	Protein A	0.03	−0.11

a Pearson's *r* between microneutralization titer against A/New York/108/2016 (H7N2) and MFI values against recombinant ectodomain (Ecto) and/or globular head domain HA1 (GH HA1).

b Pearson's *r* between microneutralization titer against A/duck/Bangladesh/19097/2013 (H5N1, 2.3.2.1a) and MFI values against recombinant ectodomain (Ecto) and/or globular head domain HA1 (GH HA1).

c The highest Pearson's *r* was shown in bold.

## 2 MATERIALS AND METHODS

### 2.1 Human sera

Three sets of human serum samples were used in this study (Tables 2 and S1). In the A(H7N2) study, single convalescent serum specimens (S2) were collected from 119 shelter workers and volunteers (18‐73 years, median age 31 years) during an A(H7N2) animal shelter outbreak in New York City in 2016‐2017, and the median days from last exposure to serum collection was 36 days (Table 2). One seropositive participant (MN titers ≥40 and HI titers ≥40), 5 indeterminate participants (MN titer ≥40 and HI titer < 40), and 113 seronegative participants (MN < 40 and HI < 40) were identified in the sero‐epidemiology study.^7^

**Table 2:** Serum samples tested by Mock, 2‐Ads, AND 8‐Ads in this study

Serum source	Novel virus exposure	Total				Mock^a^			2‐Ads^b^			8‐Ads^c^		
		S1	S2	Sample number	Median age (range)	Sample number	MN ≥ 40 n (GMT)^d^	MN < 40 n (GMT)^e^	Sample number	MN ≥ 40 n (GMT)^d^	MN < 40 n (GMT)^e^	Sample number	MN ≥ 40 n (GMT)^d^	MN < 40 n (GMT)^e^
New York City	A(H7N2)	N/A	119^f^	119^f^	31 (18‐73)	119^f^	6 (50)	113 (7)	18^g^	6 (50)	12 (11)	15^h^	6 (50)	9 (14)
Bangladesh	A(H5N1) and other avian viruses	29^i^	29^j^	58^k^	23 (16‐76)	58^k^	12 (48)	48 (9)	14^l^	5 (51)	9 (18)	9^m^	4 (48)	5 (23)

Note

and four rHAs from H3N2 (A/Bangkok/1/79, A/Beijing/32/92, A/Perth/16/2009, and A/Maryland/26/2014).

a Mock‐treated samples.

b Two ectodomain rHAs from A/California/7/2009 (H1N1) and A/Perth/16/2009 (H3N2) were used in serum adsorption.

c Total eight ectodomain rHAs were used in serum adsorption, four rHAs from H1N1 (A/USSR/90/77, A/Taiwan/01/86, A/New Caledonia/20/99, and A/California/7/2009).

d Serum sample number and MN titer GMT were shown that showed MN ≥ 40 against A/New York/108/2016 (H7N2) or A/duck/Bangladesh/19097/2013.

e Serum sample number and MN titer GMT were shown that showed MN < 40 against A/New York/108/2016 (H7N2) or A/duck/Bangladesh/19097/2013.

f Convalescent sera (S2) collected from animal shelter workers and volunteers during A(H7N2) outbreak in cat in New York 2016‐2017.

g Convalescent sera (S2) that showed MFI against ectodomain HA from A/New York/108/2016 (H7N2) more than 3500, except one serum showed MN ≥ 40, MFI = 2239.

h Convalescent sera (S2) that showed MFI against ectodomain HA from A/New York/108/2016 (H7N2) more than 2000 after 2‐Ads, except one serum showed MN ≥ 40, MFI = 1230.

i Acute sera (S1) collected from live poultry markets in Bangladesh 2012‐2013.

j Convalescent sera (S2) were collected from live poultry markets in Bangladesh 2012‐2013.

k Acute sera (S1) and convalescent sera (S2) were collected from live poultry markets in Bangladesh 2012‐2013.

l Convalescent sera (S2) that showed MFI against globular HA1 rHA from A/Indonesia/5/2005 (H5N1) more than 1300.

m Convalescent sera (S2) that showed MFI against globular HA1 rHA from A/Indonesia/5/2005 (H5N1) more than 2000 after 2‐Ads.

In the A(H5N1) study, 29 paired acute (S1, <10 days post‐symptom onset) and convalescent (S2, 23 to 73 days post‐symptom onset) sera were collected from live poultry market workers in Bangladesh as a part of a surveillance study to identify A(H5N1) subtype influenza A virus infections (Tables 2 and S1). The interval between acute and convalescent sera ranged from 20 to 68 days. No seroconversions to A(H5N1) (A/duck/Bangladesh/19097/2013 clade 2.3.2.1a) were identified by HI and MN assays (all ≤ 2‐fold rise in HI and MN).

To investigate the antibody profiles in persons who have not been exposed to novel influenza viruses (controls), 133 anonymous human serum samples collected from States of New York and Florida during the summer seasons of 2013 and 2014 were also analyzed to determine antibody baseline by the 20‐plex assay (Table S1).

The use of sera was approved by human subject review boards of the New York City Department of Health and Mental Hygiene, International Centre for Diarrhoeal Disease Research, Bangladesh, and the Centers for Disease Control and Prevention, Atlanta, USA.

### 2.2 Twenty‐plex MAGPIX assay

Bio‐Plex Pro Magnetic COOH beads (Bio‐Rad, CA) were conjugated with nineteen trimeric ectodomain and/or GH HA1 antigens from influenza A(H1N1), A(H2N2), A(H3N2), A(H5N1), A(H7N2), A(H7N7), A(H7N9), A(H9N2), A(H13N9), B Victoria lineage (B/Brisbane/60/2008), B Yamagata lineage (B/Wisconsin/1/2010), and a protein A control as described previously (Table 1).^4^ The antigens were either in‐house made at CDC or obtained from International Reagent Resource and ThermoFisher Scientific (PA, USA) (Table S2); the purity, trimerization, and receptor‐binding activity of in‐house made rHAs were confirmed as previously described.^9^, ^10^, ^11^ The serum samples were tested at 1:40 dilution in duplicate by using in‐house made phycoerythrin‐conjugated protein A (RPE‐PA) and read by a Bio‐Plex MAGPIX Multiplex Reader. Median fluorescence intensity (MFI) was obtained and analyzed with Bio‐Plex Manager Software.^4^

### 2.3 Serum antibody adsorption by mock, two rHA adsorption (2‐Ads), or eight rHA adsorption (8‐Ads)

**Figure 1:**
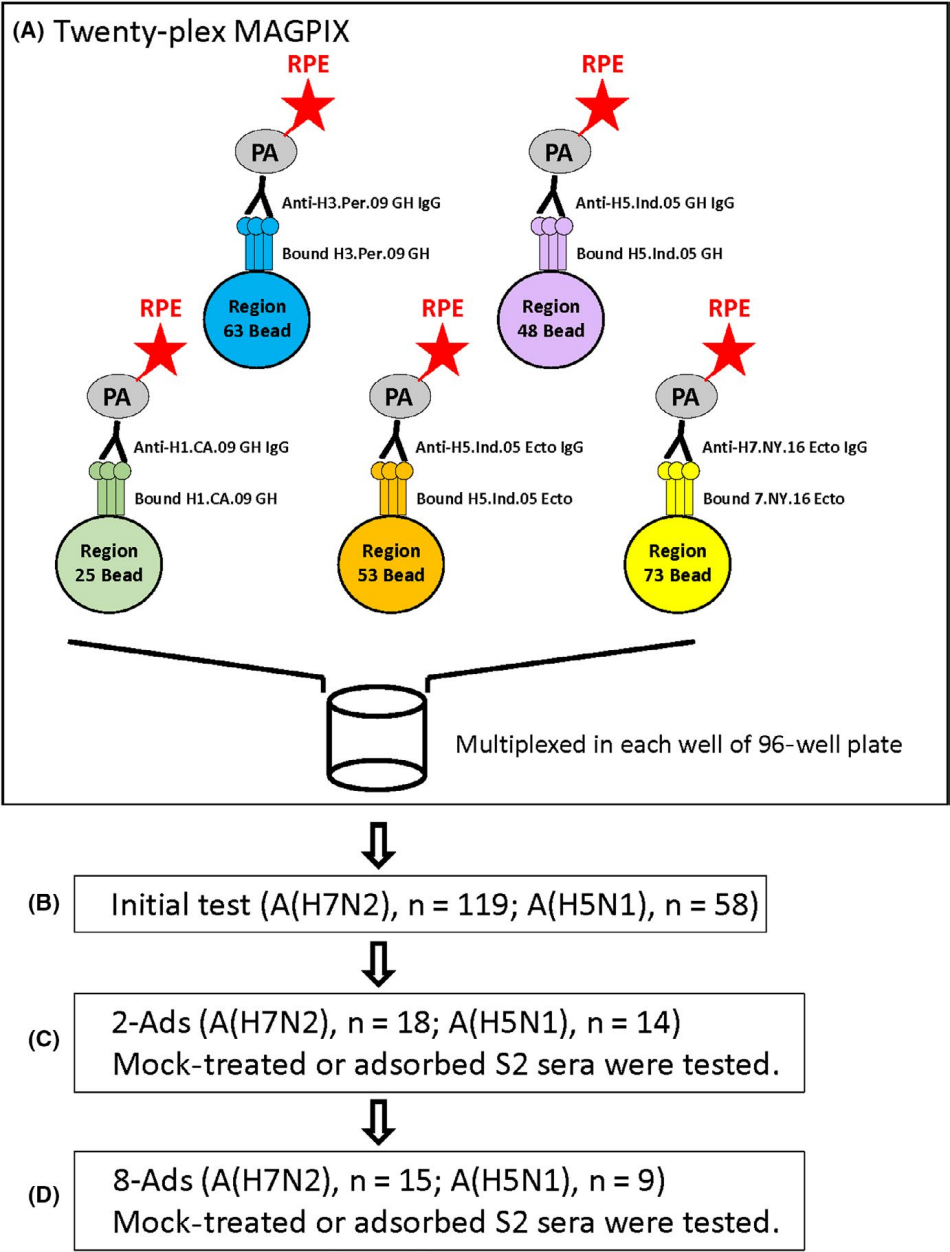
Diagram of 20‐plex MAGPIX and test flow. A, Five representative bead regions conjugated with five trimeric recombinant HAs, human anti various HA subtype antibodies, and phycoerythrin‐conjugated PA (RPE‐PA) were shown in the diagram. B, In the initial test, total 119 convalescent serum samples from A(H7N2) study and 58 serum samples (29 paired) from A(H5N1) study were tested by 20‐plex MAGPIX. C, Eighteen S2 sera from A(H7N2) study that showed MFI against H7.NY.16 Ecto more than 3500, except one serum (MFI = 2239, but MN ≥ 40), and 14 S2 sera from A(H5N1) study that showed MFI against H5.Ind.05 GH more than 1300 were retested following mock or adsorption with H1.CA.09 Ecto and H3.Per.09 Ecto‐conjugated latex beads (2‐Ads). D, Fifteen S2 sera from H7N2 study that showed MFI against H7.NY.16 Ecto more than 2000, except one serum (MFI = 1230, but MN ≥ 40), and 9 S2 sera from H5N1 study that showed MFI against H5.Ind.05 GH more than 2000 after 2‐Ads were further tested following mock or adsorption with cocktail with 4 ectodomain H1 and 4 ectodomain H3 rHA‐conjugated nickel‐coated beads (8‐Ads)

S2 samples were selected for adsorption based on MFI values (Figure S1). S2 samples that showed MFIs ≥3500 against H7.NY.16.Ecto in A(H7N2) study (except one serum showed MN ≥40 and MFI = 2239) (18 out of 119) and MFIs ≥1300 against H5.Ind.05 GH in A(H5N1) study (14 out of 29) in the initial test were first adsorbed with two ectodomain rHAs (2‐Ads) from CA/09 and Perth/09 conjugated to latex beads as described previously^4^ (Figure 1 and Table 3). Selected S2 samples for 2‐Ads showed a range of MN titers against homologous viruses from 5 to 80 (Figure S1). In 8‐Ads, either mock or adsorption with a cocktail of eight nickel‐coated magnetic beads (Thermo Fisher Scientific, PA) conjugated with each ectodomain rHA from A(H1N1) (A/USSR/90/77, A/Taiwan/1/86, A/New Caledonia/20/99, CA/09) and A(H3N2) (A/Bangkok/1/79, A/Beijing/32/92, Perth/09, and A/Maryland/26/2014) was performed as described previously.^12^ S2 specimens were tested in the 20‐plex MAGPIX platform following either mock treatment with beads only, adsorbed with 2‐Ads, or 8‐Ads (Figure 1).

**Table 3:** Ectodomain rHAs used in serum adsorption

Code	Subtype	Strain	Mock	2‐Ads	8‐Ads
H1.USSR.77 Ecto	A(H1N1)	A/USSR/90/77	−	−	+
H1.TW.86 Ecto	A(H1N1)	A/Taiwan/1/86	−	−	+
H1.NC.99 Ecto	A(H1N1)	A/New Caledonia/20/99	−	−	+
H1.CA.09 Ecto	A(H1N1)	A/California/7/2009	−	+	+
H3.BK.79 Ecto	A(H3N2)	A/Bangkok/1/79	−	−	+
H3.BJ.92 Ecto	A(H3N2)	A/Beijing/32/92	−	−	+
H3.Per.09 Ecto	A(H3N2)	A/Perth/16/2009	−	+	+
H3.MD.14 Ecto	A(H3N2)	A/Maryland/26/2014	−	−	+

Notes

“−” no rHA was used in the serum adsorption; “+” rHA was included in 2‐antigen or 8‐antigen serum adsorption.

### 2.4 HI and MN assays

Single S2 human serum samples collected from A(H7N2) study in New York City and paired S1 and S2 human serum samples collected from A(H5N1) study in Bangladesh were tested by HI and MN assays.

The modified HI assay using horse erythrocytes (1% v/v) was performed as described previously to detect antibody responses to H5 and H7 viruses.^13^ Sera were heat‐inactivated at 56°C for 30 minutes, tested for non‐specific agglutinins, and adsorbed with packed horse erythrocytes as needed. Non‐specific inhibitors in the sera were removed by incubation with receptor‐destroying enzyme at 37°C for 18‐20 hours, followed by heat inactivation prior to standard protocol,^14^ except that hemagglutination of horse erythrocytes was read after 60‐minute incubation.

MN assays were performed as described previously.^14^ Human serum samples were heat‐inactivated at 56°C for 30 minutes, and then, serial 2‐fold dilutions were made starting at an initial 1:10 dilution. Influenza viruses (one hundred of 50% tissue culture infective doses, TCID_50_) were mixed with the serum dilutions and incubated at 37°C with 5% CO_2_ for 1 hour, followed by infecting 1.5 × 10^4^ Madin**‐**Darby canine kidney (MDCK) cells per well of 96‐well plate. After 18‐hour incubation at 37°C with 5% CO_2_, viral infection was quantified by ELISA using a pool of mouse anti**‐**influenza virus A nucleoprotein (NP) monoclonal antibodies (A1 and A3, Millipore, CA). Neutralizing antibody titers were defined as the reciprocal of the highest dilution of serum samples that achieved at least 50% neutralization; geometric mean titers (GMTs) were determined from at least 2 replicates.

HI and MN assays against A/New York/108/2016 (A(H7N2) virus were performed in BSL‐2 enhanced laboratories. HI and MN assays against A/duck/Bangladesh/19097/2013 (H5N1) virus were performed in BSL‐3 enhanced laboratories.

### 2.5 Data analysis

Pearson's correlation coefficient (Pearson's *r*), paired Wilcoxon, and unpaired Mann‐Whitney test were performed using GraphPad Prism 5; *P* values of less than 0.05 were considered statistically significant.

## 3 RESULTS AND DISCUSSION

We developed a 20‐plex MAGPIX assay and expanded serum adsorption by using a cocktail of 8 rHA‐conjugated beads to reduce the effects of cross‐reactive antibodies and to improve assay performance. The platform of this 20‐plex MAGPIX, test flow, and serum adsorption is described in Figure 1. The high‐throughput MAGPIX platform can generate an antibody profile to 19 influenza antigens simultaneously by using very small volume of serum samples (<10 µL), and the similar platform showed a 4**‐**log10 linear range of sensitivity.^3^

### 3.1 Correlation between MN titers and MFIs in 20‐plex MAGPIX

Single S2 human serum samples collected from the recent New York City animal shelter A(H7N2) outbreak and paired S1 and S2 human serum samples collected from live poultry market workers in Bangladesh were analyzed by the 20‐plex MAGPIX assay in the initial test (Figure 1A,B). Typically ≥ 4‐fold rise in HI and/or MN titers from paired sera collection indicates a positive antibody response to recent influenza infection or vaccination.^1^ In the multiplex MAGPIX assay, previously we used ≥ 2‐fold rise in MFI as the cutoff to determine serologic responses from paired sera, and the influenza viruses with the highest fold rise in MFI correlated with viruses of potential exposure.^4^, ^15^ In the current study, no seroconversions (≥4‐fold rise) were detected by HI/MN for paired sera collected from A(H5N1) study. Thus, as expected, we did not observe ≥ 2‐fold rise in MFIs (data not shown).

**Figure 2:**
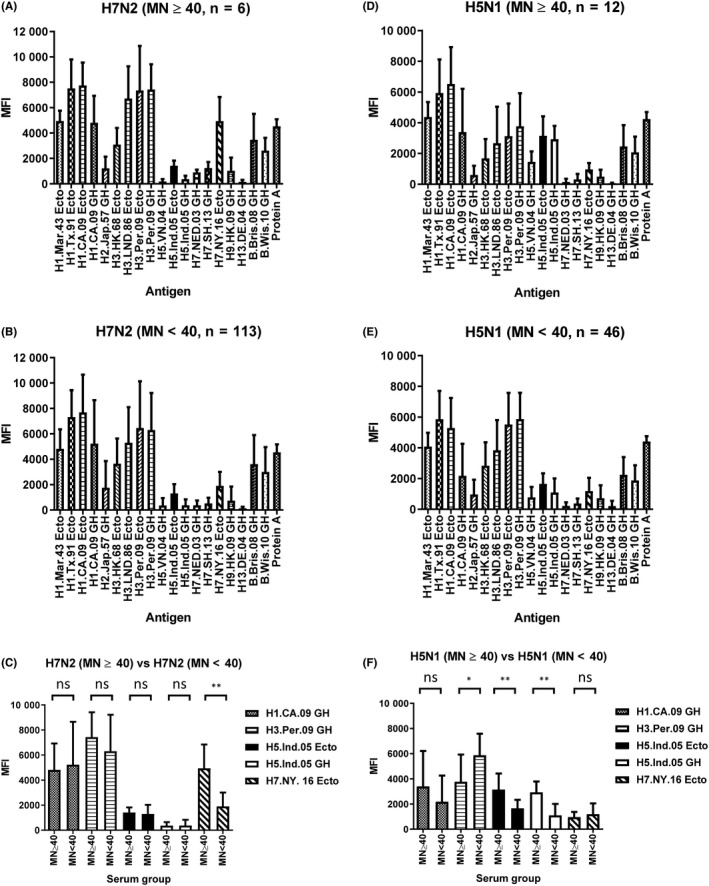
Correlations between MN titer and MFI values in the initial test. Antibody profiles of 20‐plex MAGPIX for 119 convalescent serum samples from A(H7N2) study, A (MN ≥40, n = 6) and B (MN <40, n = 113) against A/New York/108/2016, A(H7N2), and 58 serum samples from A(H5N1) study, D (MN ≥40, n = 12) and E (MN <40, n = 46) against A/duck/Bangladesh/19097/2013 (H5N1, 2.3.2.1a) were analyzed, the mean MFI value and + the standard deviation were shown. MFIs against 5 rHAs between MN ≥40 group and MN <40 group were analyzed (C, H7N2 study, F. A(H5N1) study). For each group, the mean MFI value and + the standard deviation were shown. Unpaired Mann‐Whitney tests were performed in C and F, ns: not significant (*P* ≥ .05), ^*^0.01 ≤* P *< .05, ^**^ *P *< .01

Next, we grouped S2 sera from the A(H7N2) study and both S1 and S2 serum samples from the H5N1 study based on MN titers (MN ≥40 group or MN <40 group) against exposed HA subtypes: A(H7N2) (A/New York/108/2016) or A(H5N1) virus to analyzed antibody responses measured by MFI versus MN (Figure 2A,B,D, and E). MFIs against H7.NY.16 Ecto and MFIs against H5.Ind.05 Ecto/H5.Ind.05 GH in the MN ≥40 group were significantly higher than those in the MN <40 group for the A(H7N2) and A(H5N1) studies, respectively (Figure 2C,F). Conversely, the differences between MFIs against seasonal H1, H3, and unexposed novel subtype HA were not significant (Figure 2C,F), except MFI against H3.Per.09 GH in which MFIs in the MN <40 group were higher than those in the MN ≥40 group (Figure 2F).

We also analyzed correlation between MN titers against A(H7N2) (or A(H5N1)) and MFIs against 20 antigens measured by MAGPIX (Table 1). In New York City animal shelter staff who may have had potential exposure to A(H7N2)‐infected cats, MFI against rHA from A/New York/108/2016 (H7N2) has the highest correlation to MN titers against A(H7N2) virus (Pearson's correlation coefficient *r* = 0.63, n = 119, Table 1). Similarly, for Bangladesh poultry workers who may have been exposed to A(H5N1), the GH HA1 and ectodomain rHA from A/Indonesia/05/2005 (A(H5N1)) had the highest correlation with MN titers against A(H5N1) (Pearson's correlation coefficient *r* = 0.78, n = 58, Table 1). We also analyzed the correlation between MN titers and MFIs against H7 and H5 rHAs for each MN titer group (MN ≥40, MN <40, or all samples; Pearson's *r* values ranged from 0.45 to 0.78, Figure S2). These results suggest that among the antibody profiles to the 19 HAs (from 11 influenza subtypes) measured by MAGPIX, MFI against rHA from the exposed novel HA subtype had the highest correlation to MN titers to the same virus. It is consistent with our previous observation during the first wave of A(H1N1)pdm09 pandemic.^16^

### 3.2 Serum adsorption by using either 2 or 8 rHAs from A(H1N1) and A(H3N2) to reduce cross‐reactivity and improve the detection of subtype‐specific responses to novel HA subtypes

Complex exposures to seasonal influenza virus(es) and/or vaccine(s) induce both within‐subtype (homosubtypic) and cross‐subtype (heterosubtypic) reactivity. The presence of cross‐reactive antibodies against unexposed subtype HA has been described when ectodomain HAs are used.^16^, ^17^ The use of GH HA1 rHAs that lack the antigenically conserved HA stalk can reduce such cross‐reactivity.^18^ In this study, we observed high MFIs against H5.Ind.05 Ecto in specimens collected from the A(H7N2) serosurvey in New York City, where participants have never had A(H5N1) virus exposures (Figure 2A‐C). Likewise, some poultry workers from the A(H5N1) serosurvey also demonstrated high MFIs against H7.NY.16 Ecto (Figure 2D‐F). This suggests that novel influenza viruses may share common epitopes that can be detected by binding assays.^4^ Although cross‐reactive antibody responses can be beneficial in providing heterologous protections in vaccine design, here in serologic diagnosis, antibody cross‐reactivity poses challenges when serology is used to assess subtype‐specific exposure to novel influenza viruses, especially, when the samples are collected from areas where multiple subtypes and strains of avian influenza viruses co‐circulate.^4^

**Figure 3:**
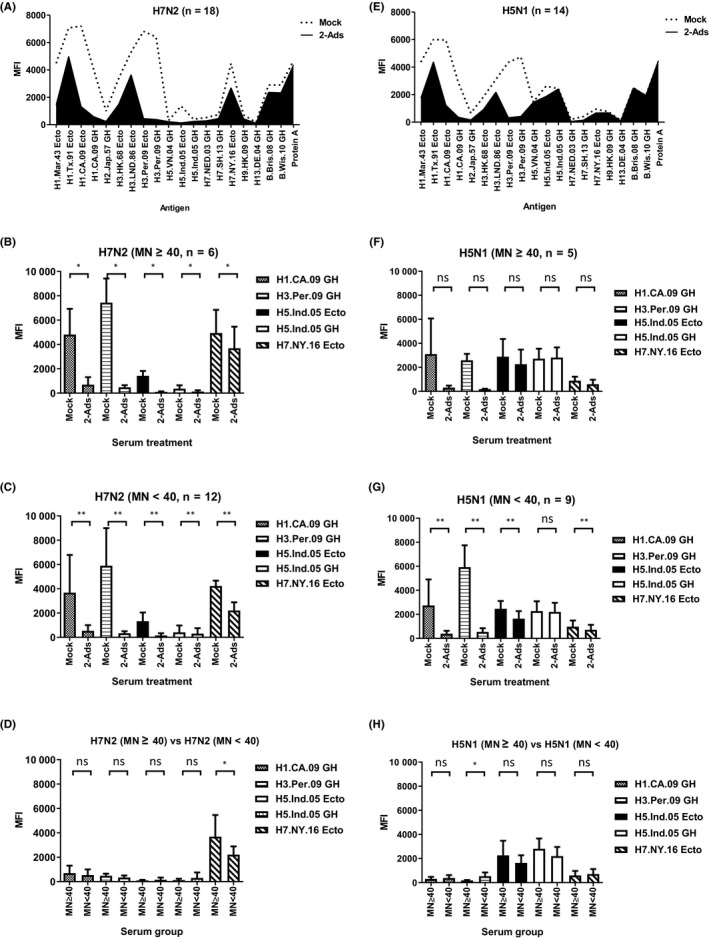
Antibody profiles were changed following mock or 2‐Ads. Analysis of antibody profiles of 18 S2 samples that showed high MFIs against H7.NY.16 Ecto from A(H7N2) study (A) or 14 S2 samples that showed high MFIs against H5.Ind.05 GH from A(H5N1) study (E) following mock or latex beads conjugated with H1.CA.09 Ecto and H3.Per.09 Ecto (2‐Ads). The statistical analysis of MFIs against 5 rHAs was performed for mock‐ or 2‐Ads‐treated samples. B, A(H7N2) study, MN ≥40 (n = 6), C, A(H7N2) study, MN <40 (n = 12). F. A(H5N1) study, MN ≥40 (n = 5); G, A(H5N1) study, MN <40 (n = 9). MFIs between serum group MN ≥40 and serum group MN <40 following 2‐Ads were analyzed (D, A(H7N2), H. A(H5N1)). For each group, the mean MFI value and + the standard deviation were shown. Paired Wilcoxon (B, C, F, and G) and unpaired Mann‐Whitney tests were performed, ns: not significant (*P* ≥ .05), ^*^0.01 ≤ *P *< .05, ^**^ *P *< .01

We have previously demonstrated that cross‐reactivity could be reduced by incorporating a serum adsorption step prior to the MAGPIX or MN assays, and the remaining signals from post‐adsorption samples represent HA subtype‐specific responses.^4^, ^7^ Thus, serum adsorptions were performed in the current study to remove cross‐reactive antibodies to improve sensitivity and specificity. Following the initial analysis, eighteen S2 samples from the H7N2 study that showed either MN ≥40 or MFI >3500 against H7.NY.16 Ecto (H7N2) and 14 samples that showed MFI >1300 against H5.Ind.05 GH (H5N1, clade 2.1.3.2) from H5N1 study were selected for either mock or adsorption using two rHAs (2‐Ads) (Figure 1C, Tables 2 and 3, and Figure S1). After 2‐Ads, antibody profiles shifted dramatically, and MFIs against cross‐reactive epitopes from rHAs in H1.CA.09 GH and H3.Per.09 GH were reduced to background level (Figure 3A,E), including MFIs against H5.Ind.05 for serum samples collected in the H7N2 study (Figure 3A).

We also analyzed MFIs after grouping adsorbed S2 samples from the A(H7N2) study based on MN titer against A(H7N2) (MN ≥40 and MN <40), as shown in Figure 3B,C, MFIs against selected rHAs in both groups were significantly reduced after 2‐Ads compared to mock‐treated samples, but MFIs to H7.NY.16 Ecto mostly remained. On the other hand, in the S2 sera collected from poultry workers exposed to A(H5N1), MFIs against seasonal H1 and H3 were reduced for both the MN ≥40 and MN <40 groups after 2‐Ads (Figure 3F,G), but only MN <40 group reached statistically significant difference (Figure 3G). MFIs against H5.Ind.05 Ecto and H7.NY.16 Ecto domain rHAs in the MN <40 group were significantly reduced after 2‐Ads, but not for antibodies against H5.Ind.05 GH (Figure 3G), suggesting antibodies against GH HA1 are more strain‐specific, while antibodies against the whole ectodomain containing the HA stalk region are more cross‐reactive.^18^, ^19^

The effects of 2‐Ads between the MN ≥40 and MN <40 groups were also analyzed, MFIs against H7.NY.16 Ecto in the MN ≥40 group from the sera collected in the A(H7N2) study were significantly higher than those in MN <40 group (*P* < .05, Figure 3D), and it was consistent with the results from mock‐treated samples (Figure 2C). MFIs against H3.Per.09 GH in the MN <40 group from A(H5N1) study were significantly higher than those in the MN ≥40 group for Mock or 2‐Ads (Figure 2F and 3H). On the other hand, MFIs against H5.Ind.05 were not significantly different after 2‐Ads (Figure 3H).

**Figure 4:**
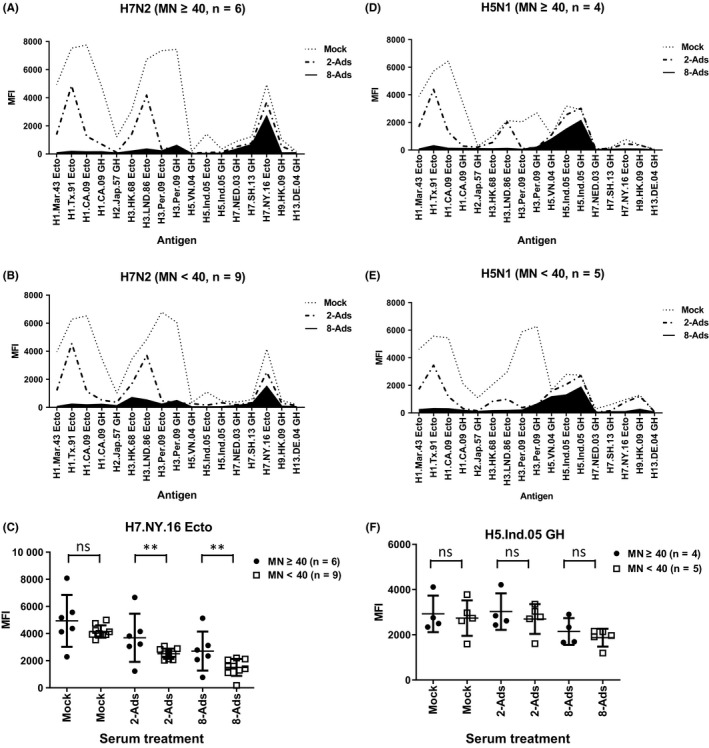
MFIs against H1 and H3 rHAs were removed by 8‐Ads. Fifteen S2 samples that showed high MFIs against H7.NY.16 Ecto from A(H7N2) study or 9 S2 samples that showed high MFIs against H5.Ind.05 GH from A(H5N1) study after 2‐Ads were further adsorbed by either mock, 2‐Ads, or cocktail of 8 rHA‐conjugated nickel‐coated beads (8‐Ads). The treated samples were tested by 20‐plex MAGPIX, A, (A(H7N2), MN ≥40, n = 6), B, (A(H7N2), MN <40, n = 9), D, (A(H5N1), MN ≥40, n = 4), and E, (A(H5N1), MN <40, n = 5). MFIs between serum group MN ≥40 and serum group MN <40 following mock, 2‐Ads, or 8‐Ads were analyzed (C, H7N2, F. H5N1). For each group, the mean MFI value and + the standard deviation were shown. Unpaired Mann‐Whitney tests were performed in C and F, ns: not significant (*P* ≥ .05), ^*^0.01 ≤ *P *< .05, ^**^ *P *< .01

Of note, even after 2‐Ads, MFIs against some seasonal H1 and H3 are still detectable (Figure 3A,E), suggesting cross‐reactive antibodies could still remain, reflecting the complex exposure history to multiple seasonal influenza viruses in these individuals. Three S2 sera from A(H7N2) study showed more than 60% reduction of MFI against H7.NY.16 Ecto after 2‐Ads, they were excluded in 8‐Ads, and on the other hand, only one S2 serum from A(H5N1) study showed 21% reduction of MFI against H5.Ind.05 GH (Figure 3E‐G, and data not shown). To further reduce cross‐reactive antibodies against seasonal H1 and H3 HAs, we expanded the adsorption with a cocktail of eight ectodomain HAs (8‐Ads) (Table 3). We selected the 6 additional rHAs for adsorption from viruses representative of antigenic clusters of seasonal A(H1N1)^20^ and A(H3N2) viruses (Table 3) that the population may have been exposed to. Fifteen S2 samples from A(H7N2) study that showed either MN ≥40 or MFI >2000 against H7.NY.16 Ecto from A(H7N2) study and 9 out of 14 S2 samples that showed either MN ≥40 or MFI >2000 against H5.Ind.05 GH (H5N1, clade 2.1.3.2) from the A(H5N1) study after 2‐Ads were further treated by 8‐Ads (Tables 2 and 3, and Figure 1D). To this end, following 8‐Ads, most cross‐reactive antibodies were removed, with MFIs to unexposed HAs reduced to almost background level (MFI <500) for sera from both the A(H7N2) and A(H5N1) studies, MFI value less than 500 was considered as “background” in our current platform^4^; however, the strain‐specific signals against potential exposed H7 and H5 HAs remained at similar levels (Figure 4A,B,D, and E).

The difference between the MN ≥40 and MN <40 groups was analyzed after adsorption. For sera collected from the A(H7N2) study, MFIs against H7.NY.16 Ecto in MN ≥40 were significantly higher than those in the MN <40 group after 2‐Ads and 8‐Ads (Figure 4C). Serum adsorption reduced cross‐reactive binding antibodies and enhanced the difference between the MN ≥40 and MN <40 groups for samples from the A(H7N2) study. The results are consistent with our previous report, and exposed novel HA antigen can be determined more accurately after serum adsorption.^4^, ^7^ On the other hand, no significant difference was achieved for samples from the A(H5N1) study, partly due to the selection of samples that showed either high cross‐reactivity in MFIs against H5.Indo.06 GH after 2‐Ads (>2000) and/or relatively high MN titer against A/duck/Bangladesh/1907/2013 (GMT 23, Table 2). In this study, since we performed 2‐Ads and 8‐Ads by using cocktailed H1 (group 1) and H3 (group 2) rHAs, we did observe some reductions in MFIs against exposed novel subtype HAs (Figure 3 and 4). It will be interesting to perform HA group‐specific rHA adsorption in the future studies.

### 3.3 Correlation between age and MFIs against rHAs from various subtype influenza viruses

**Figure 5:**
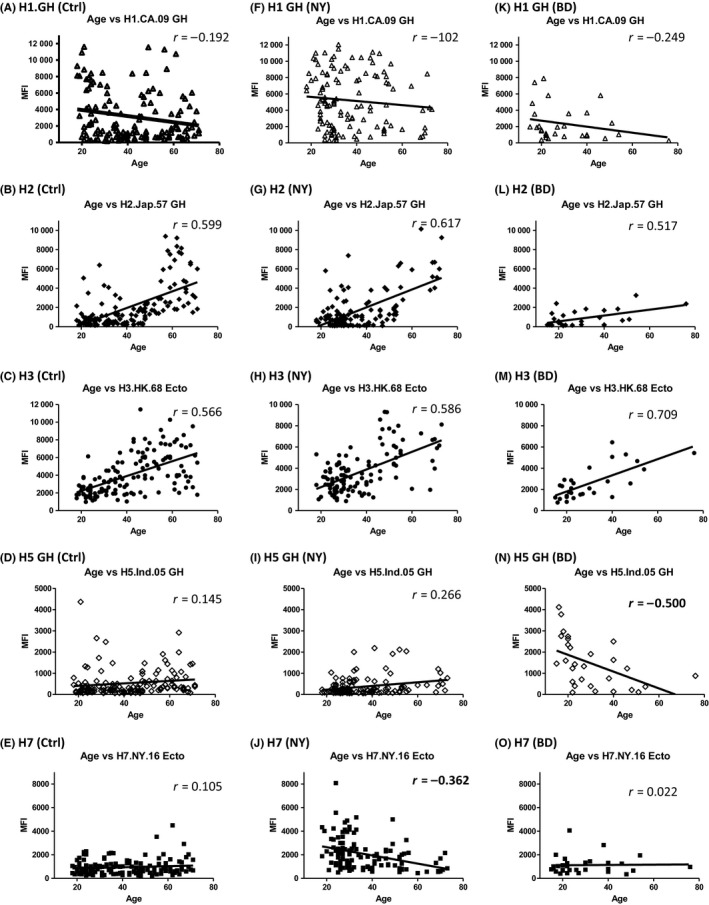
Different Pearson's correlation coefficients between age and MFI were achieved. The correlation between ages of age‐matched controls (Ctrl) (A, B, C, D, and E), 119 subjects in A(H7N2) New York study (NY) (F, G, H, I, and J), or 29 subjects in A(H5N1) Bangladesh (BD) study (K, L, M, N, and O) and MFIs against selected rHAs were analyzed. H1.CA.09 Ecto (A, F, and K); H2.Jap.57 GH (B, G, and L); H3.HK.68 Ecto (C, H, and M); H5.Ind.05 GH (D, I, and N); H7.NY.16 Ecto (E, J, and O)

Exposures to multiple influenza HA antigens throughout an individual's life span can result in both HA subtype‐specific and cross‐reactive antibodies, and the presence of cross‐reactive antibodies often complicates the interpretation of serologic data.^4^ The baseline antibodies to novel subtype influenza viruses can reflect existing population immunity to novel viruses and are important parameters for influenza risk assessment. Baseline antibody profiles of the population vary by age and exposure history to seasonal and novel influenza viruses. We analyzed the correlation between age and antibody levels to multiple influenza HAs measured by MAGPIX. We included a set of age‐matched normal human serum samples collected from the States of New York and Florida during the summer seasons of 2013 and 2014 when there was no seasonal influenza virus circulation as a baseline control (Table S1). Age and MFIs against rHA from H2 and an early H3 (H3.HK.68 Ecto) (two antigens are no longer circulating) were positively correlated (Pearson's correlation coefficient *r*> 0.5) in A(H7N2) and A(H5N1) studies (Figure 5B,C,G,H,L,M, Table S3), because older persons have likely been exposed to H2 and early H3 influenza viruses.^4^ Together with findings from other studies, the results suggest that age‐matched controls are often necessary to determine baseline levels of population immunity in studies of different birth cohorts.^4^, ^18^, ^21^, ^22^

Interestingly, we found that there was a negative correlation (negative Pearson's *r* value) between age and exposed novel HA antigen H7.NY.16 Ecto for New York A(H7N2) study (*r* = −0.362, Figure 5J), in contrast to positive (*r* = 0.105, Figure 5E) and low Pearson's correlation coefficient (*r* = 0.022, Figure 5O) for age‐matched control sera and sera from the Bangladesh A(H5N1) study, respectively (Table S3). Similarly, there was also a negative correlation (negative Pearson's *r* value) between age and exposed H5 antigens for sera collected in the Bangladesh A(H5N1) study (*r* = −0.500, Table S3), in contrast to positive correlation for age‐matched control sera (*r* = 0.145, Figure 5D) and for animal shelter staff in the New York A(H7N2) study (*r* = 0.266, Figure 5I and Table S3). Similarly, negative correlations between age and MN titer against A(H7N2) (Pearson's *r* value = −0.20) or A(H5N1) (Pearson's *r* value = −0.31) were observed (Figure S3).

In general, antibodies to novel virus HA in unexposed populations increase with age and vary by geographical location due to cross‐reactivity between the novel virus and seasonal influenza viruses through past exposure.^18^, ^21^, ^23^, ^24^, ^25^, ^26^ When A(H1N1)pdm09 virus was first introduced to the human population in 2009, higher MN titers were observed in older age groups.^25^ Here, low negative Pearson's *r* values between age and MFI against H1.CA.09 were observed (−0.102 to −0.249) for sera from age‐matched controls, A(H7N2), and A(H5N1) studies, all collected several years post‐2009 pandemic (Figure 5A,F,K), indicating exposures to A(H1N1)pdm09 influenza viruses through infections/vaccines that occurred across all ages of the population since 2009.^27^, ^28^ Further, when sera collected in the A(H7N2) study in 2016‐2017 were analyzed for MN titers against A(H1N1)pdm09, negative correlation between age and A(H1N1)pdm09 MN titers was also observed (Figure S4) and was consistent with the results from MAGPIX (Figure 5F).

Our results suggest that analysis of correlation between age and MFIs against multiple subtype HAs can provide important information to determine potential exposures to novel subtype HA influenza viruses in a sub‐population. This also suggests that age‐matched controls selected from the same region and time period are necessary for proper analysis of MAGPIX results (Figure 5, Tables S1 and S3). For example, the age‐related antibody baseline level to A(H1N1)pdm09 in recent years would be much higher than those reported in 2009,^29^ due to extensive exposure to A(H1N1)pdm09 virus through infection or vaccination post‐2009.^16^ If we can determine whether low levels of age‐specific, subtype cross‐reactive antibodies are present in the population, single convalescent‐phase serum specimens may be sufficient to determine a recent exposure to novel subtype influenza.^7^, ^29^

Our study has several limitations: (a) Given the availability of only small numbers of sera, we could not determine cutoff value for potential exposure to novel subtype HA for a given population; (b) without paired sera samples demonstrating sera conversion, we could not rule out the possibility of exposure to novel subtype influenza virus(es) from past infections rather than the current outbreak.

The twenty‐plex MAGPIX assay combined with 8‐Ads and analysis of correlation between age and MFIs can be used to screen potential exposed novel HA subtype virus for further analysis. This high‐throughput platform will streamline serologic analysis of human or animal serum samples that are collected from areas where multiple novel HA subtype viruses co‐circulate. This platform can be further expanded to incorporate influenza virus rHAs from all 18 HA subtypes, which could be used to determine exposures to novel influenza viruses in humans, or to identify new influenza virus host using animal sera instead of labor‐intensive ELISA.^30^

In summary, the 20‐plex MAGPIX assay combined with 8‐Ads and correlation analysis between age and MFIs will be a useful tool to identify exposure to novel HA subtype influenza viruses in high‐risk populations, especially when rRT‐PCR, virus isolation, or well‐paired sera are not available.

## 4 CONFLICT OF INTEREST STATEMENT

All other authors report no potential conflicts. Conflicts that the editors consider relevant to the content of the manuscript have been disclosed.

## 5 DISCLAIMER

Trade names are only used for identification, and they are not endorsed by the Public Health Service or by the US Department of Health and Human Services. The findings and conclusions in this report are those of the authors and do not necessarily represent the official position of CDC.
