# Supplementary material for: Identification of novel influenza A virus exposures by an improved high‐throughput multiplex MAGPIX platform and serum adsorption
Source: Influenza Other Respir Viruses. 2019 Nov 8;14(2):129–41. doi: 10.1111/irv.12695 (PMC7040970; doi:10.1111/irv.12695)
Supplement: Supplementary file 1 [file IRV-14-129-s001.pptx]

## Slide 1
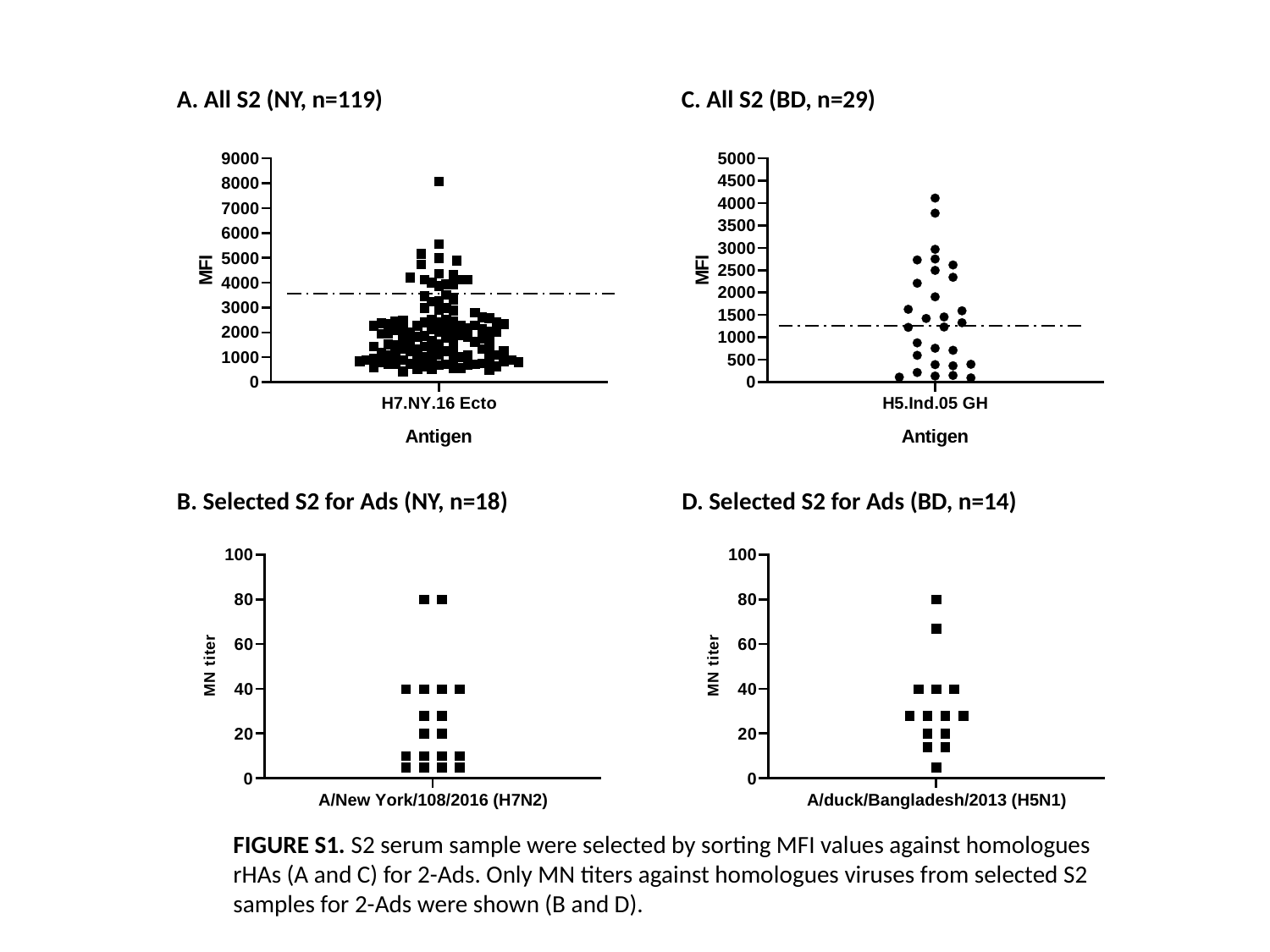

A. All S2 (NY, n=119)
C. All S2 (BD, n=29)
B. Selected S2 for Ads (NY, n=18)
D. Selected S2 for Ads (BD, n=14)
FIGURE S1. S2 serum sample were selected by sorting MFI values against homologues rHAs (A and C) for 2-Ads. Only MN titers against homologues viruses from selected S2 samples for 2-Ads were shown (B and D).
