# Supplementary material for: Identification of novel influenza A virus exposures by an improved high‐throughput multiplex MAGPIX platform and serum adsorption
Source: Influenza Other Respir Viruses. 2019 Nov 8;14(2):129–41. doi: 10.1111/irv.12695 (PMC7040970; doi:10.1111/irv.12695)
Supplement: Supplementary file 2 [file IRV-14-129-s002.pptx]

## Slide 1
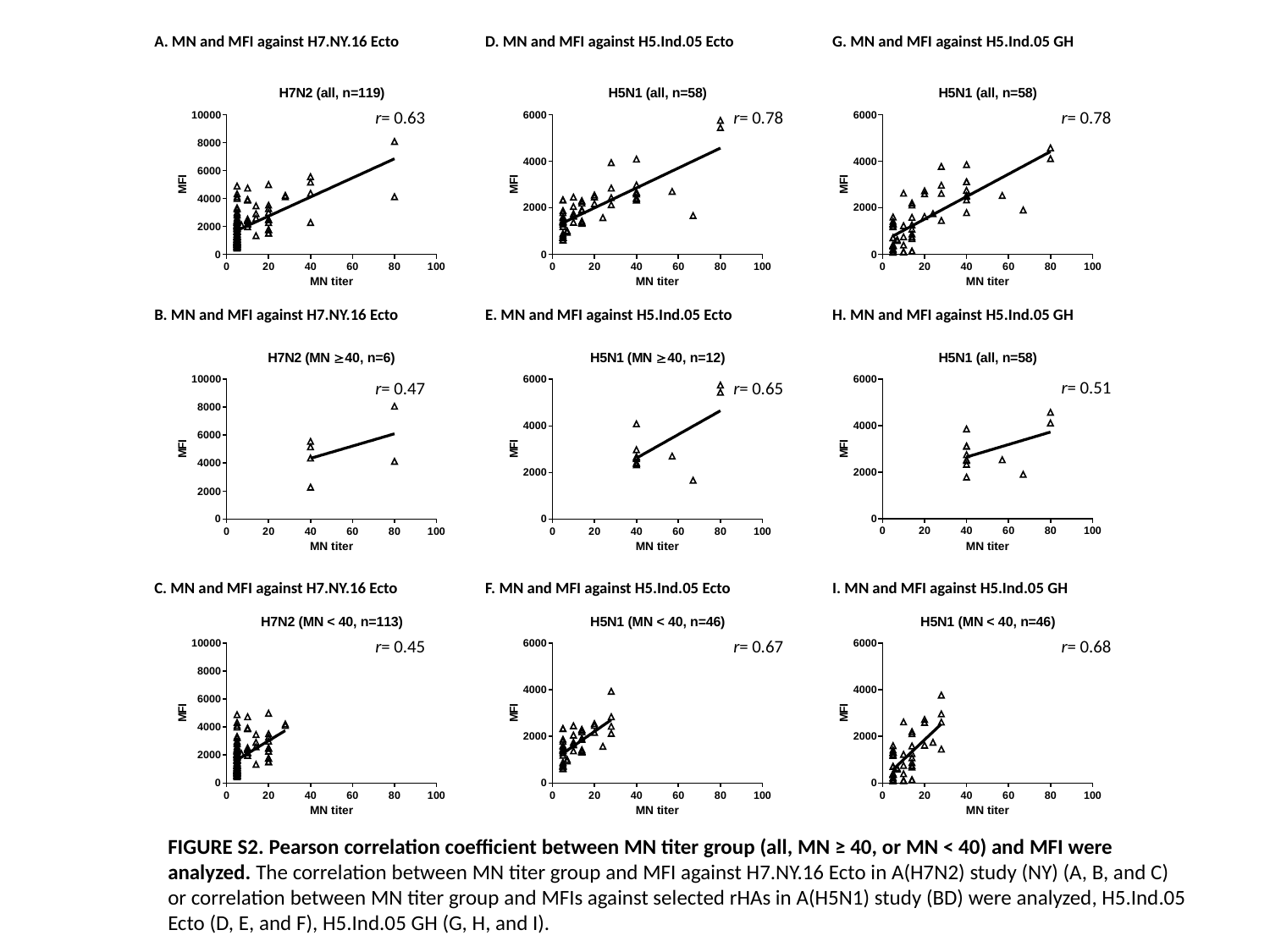

A. MN and MFI against H7.NY.16 Ecto
D. MN and MFI against H5.Ind.05 Ecto
G. MN and MFI against H5.Ind.05 GH
r= 0.63
r= 0.78
r= 0.78
B. MN and MFI against H7.NY.16 Ecto
E. MN and MFI against H5.Ind.05 Ecto
H. MN and MFI against H5.Ind.05 GH
r= 0.51
r= 0.47
r= 0.65
C. MN and MFI against H7.NY.16 Ecto
F. MN and MFI against H5.Ind.05 Ecto
I. MN and MFI against H5.Ind.05 GH
r= 0.45
r= 0.67
r= 0.68
FIGURE S2. Pearson correlation coefficient between MN titer group (all, MN ≥ 40, or MN < 40) and MFI were analyzed. The correlation between MN titer group and MFI against H7.NY.16 Ecto in A(H7N2) study (NY) (A, B, and C) or correlation between MN titer group and MFIs against selected rHAs in A(H5N1) study (BD) were analyzed, H5.Ind.05 Ecto (D, E, and F), H5.Ind.05 GH (G, H, and I).
