# Supplementary material for: Identification of novel influenza A virus exposures by an improved high‐throughput multiplex MAGPIX platform and serum adsorption
Source: Influenza Other Respir Viruses. 2019 Nov 8;14(2):129–41. doi: 10.1111/irv.12695 (PMC7040970; doi:10.1111/irv.12695)
Supplement: Supplementary file 3 [file IRV-14-129-s003.pptx]

## Slide 1
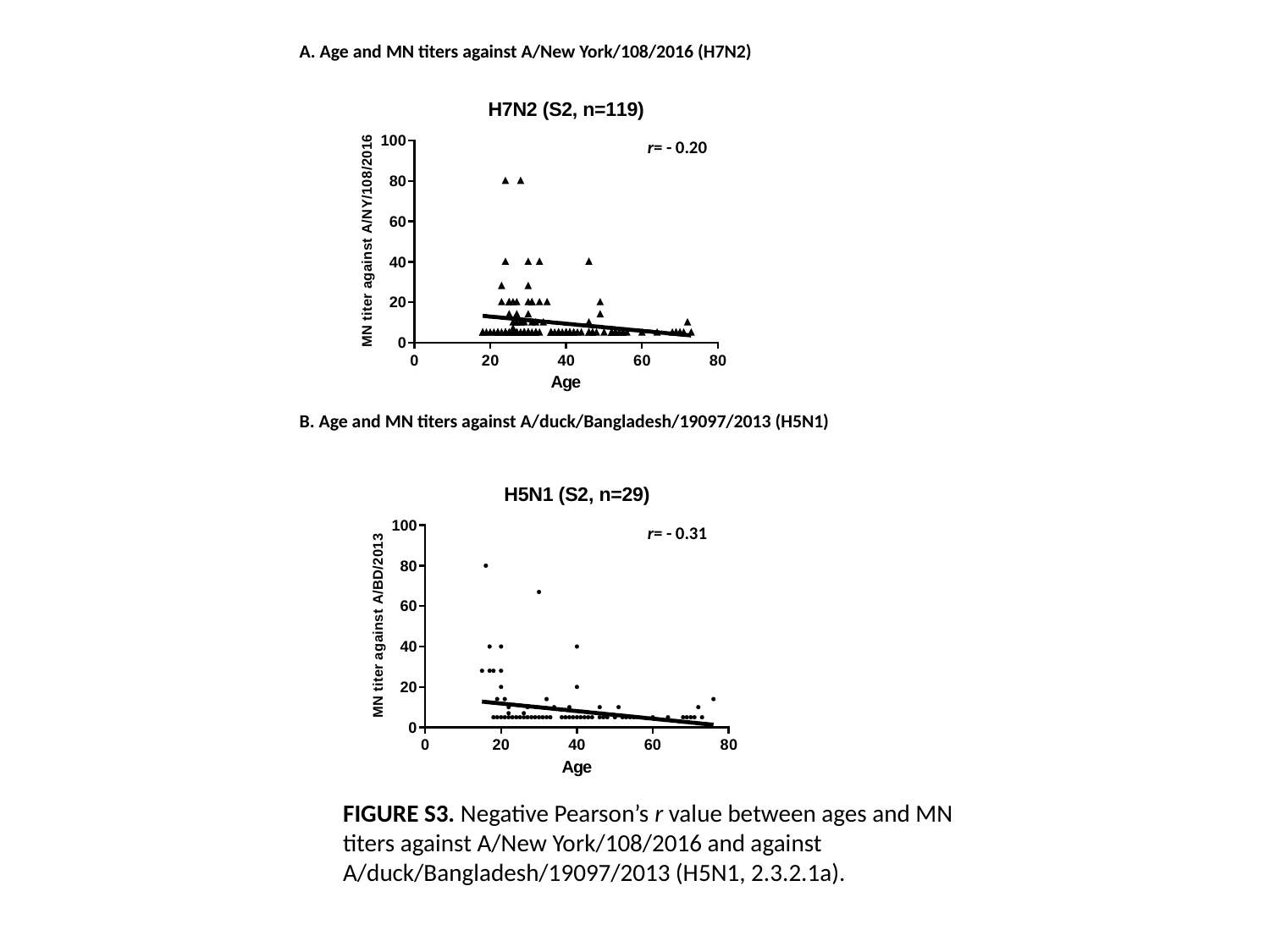

A. Age and MN titers against A/New York/108/2016 (H7N2)
r= - 0.20
B. Age and MN titers against A/duck/Bangladesh/19097/2013 (H5N1)
r= - 0.31
FIGURE S3. Negative Pearson’s r value between ages and MN titers against A/New York/108/2016 and against A/duck/Bangladesh/19097/2013 (H5N1, 2.3.2.1a).
