# Supplementary material for: Identification of novel influenza A virus exposures by an improved high‐throughput multiplex MAGPIX platform and serum adsorption
Source: Influenza Other Respir Viruses. 2019 Nov 8;14(2):129–41. doi: 10.1111/irv.12695 (PMC7040970; doi:10.1111/irv.12695)
Supplement: Supplementary file 4 [file IRV-14-129-s004.pptx]

## Slide 1
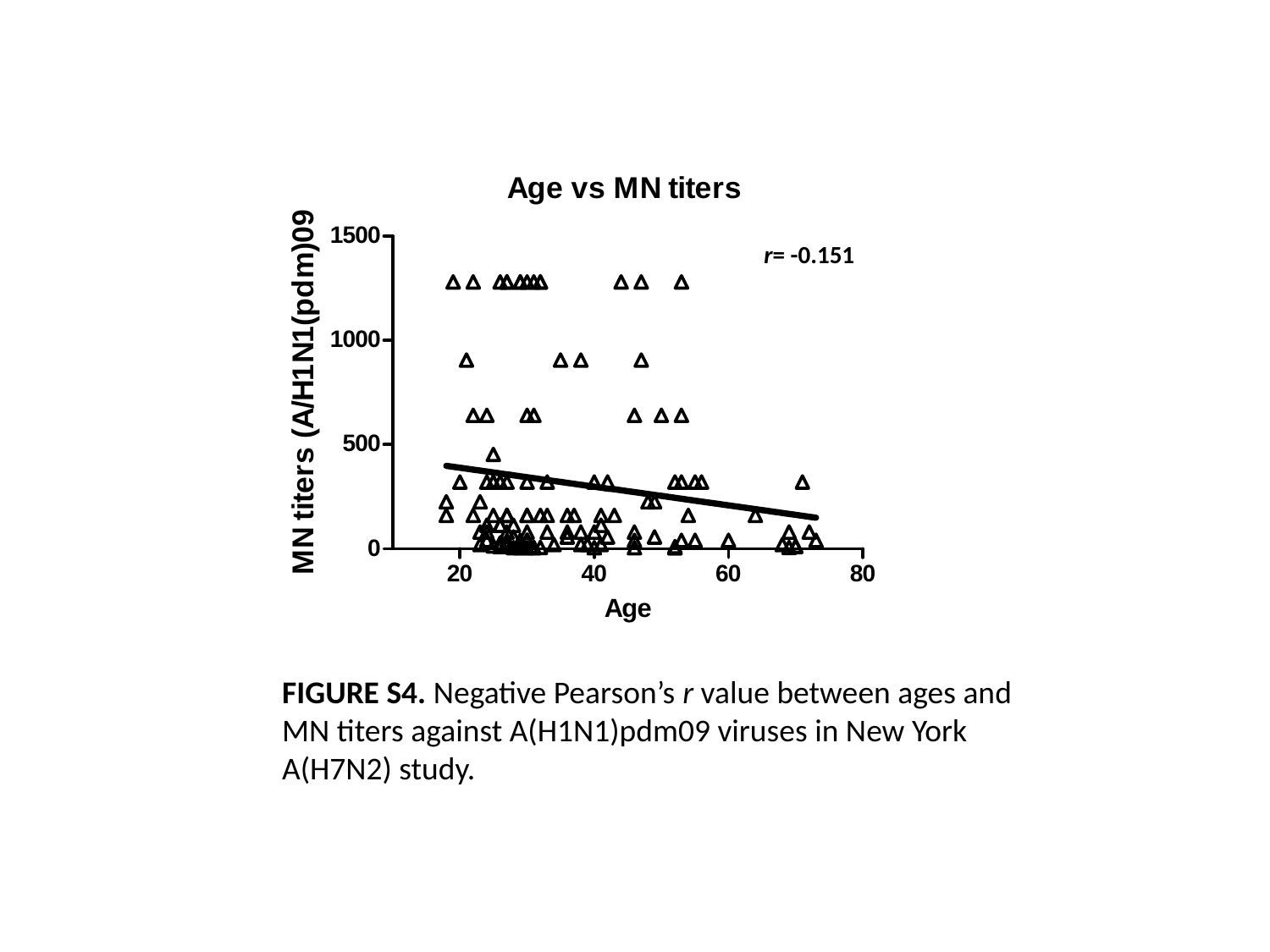

r= -0.151
FIGURE S4. Negative Pearson’s r value between ages and MN titers against A(H1N1)pdm09 viruses in New York A(H7N2) study.
