# Supplementary material for: Identification of novel influenza A virus exposures by an improved high‐throughput multiplex MAGPIX platform and serum adsorption
Source: Influenza Other Respir Viruses. 2019 Nov 8;14(2):129–41. doi: 10.1111/irv.12695 (PMC7040970; doi:10.1111/irv.12695)
Supplement: Supplementary file 5 [file IRV-14-129-s005.pptx]

## Slide 1
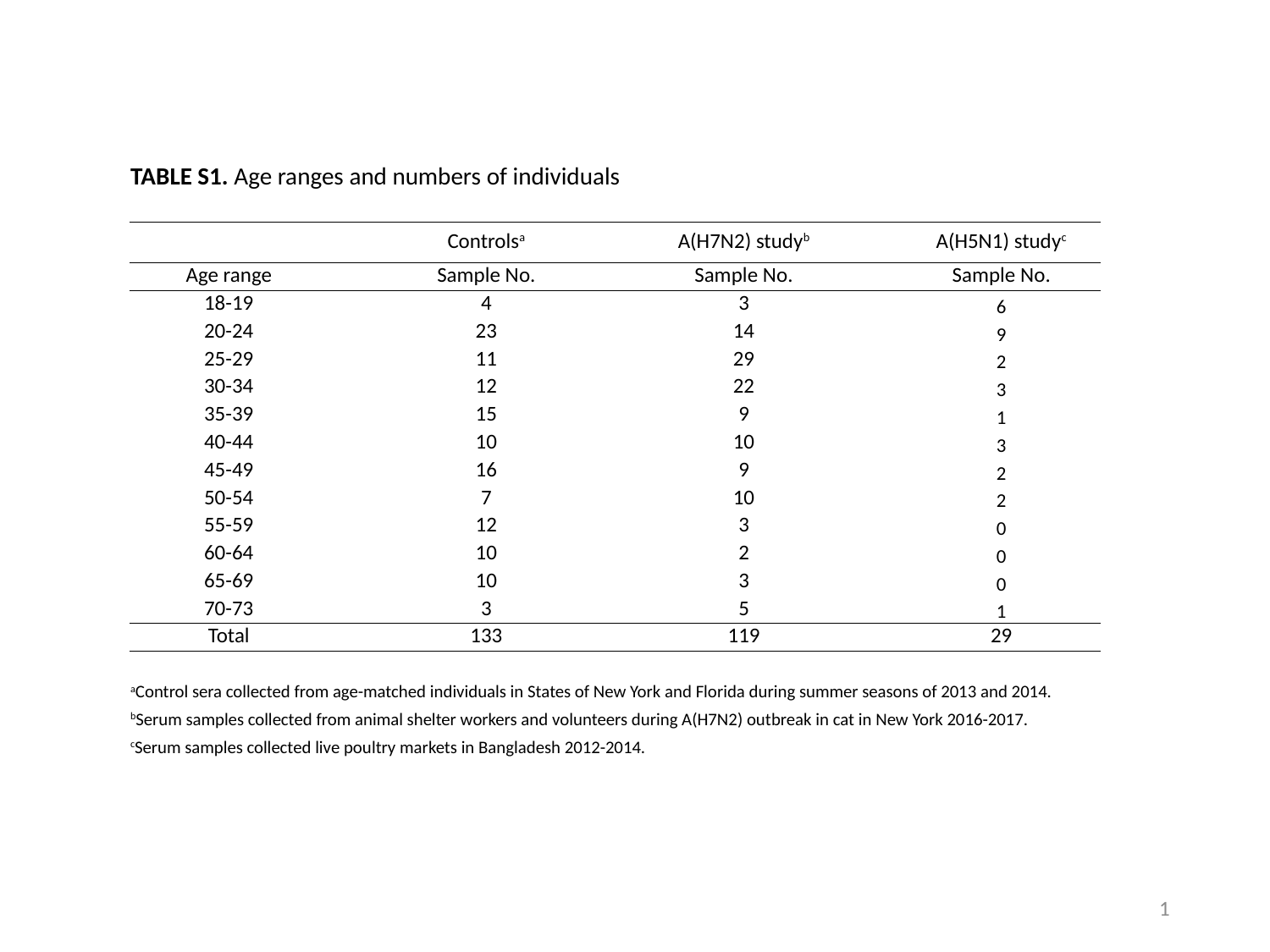

| TABLE S1. Age ranges and numbers of individuals | | | | | | |
| --- | --- | --- | --- | --- | --- | --- |
| | | | | | | |
| | | Controlsa | | A(H7N2) studyb | | A(H5N1) studyc |
| Age range | | Sample No. | | Sample No. | | Sample No. |
| 18-19 | | 4 | | 3 | | 6 |
| 20-24 | | 23 | | 14 | | 9 |
| 25-29 | | 11 | | 29 | | 2 |
| 30-34 | | 12 | | 22 | | 3 |
| 35-39 | | 15 | | 9 | | 1 |
| 40-44 | | 10 | | 10 | | 3 |
| 45-49 | | 16 | | 9 | | 2 |
| 50-54 | | 7 | | 10 | | 2 |
| 55-59 | | 12 | | 3 | | 0 |
| 60-64 | | 10 | | 2 | | 0 |
| 65-69 | | 10 | | 3 | | 0 |
| 70-73 | | 3 | | 5 | | 1 |
| Total | | 133 | | 119 | | 29 |
| | | | | | | |
| aControl sera collected from age-matched individuals in States of New York and Florida during summer seasons of 2013 and 2014. | | | | | | |
| bSerum samples collected from animal shelter workers and volunteers during A(H7N2) outbreak in cat in New York 2016-2017. | | | | | | |
| cSerum samples collected live poultry markets in Bangladesh 2012-2014. | | | | | | |
1
