# Supplementary material for: Identification of novel influenza A virus exposures by an improved high‐throughput multiplex MAGPIX platform and serum adsorption
Source: Influenza Other Respir Viruses. 2019 Nov 8;14(2):129–41. doi: 10.1111/irv.12695 (PMC7040970; doi:10.1111/irv.12695)
Supplement: Supplementary file 6 [file IRV-14-129-s006.pptx]

## Slide 1
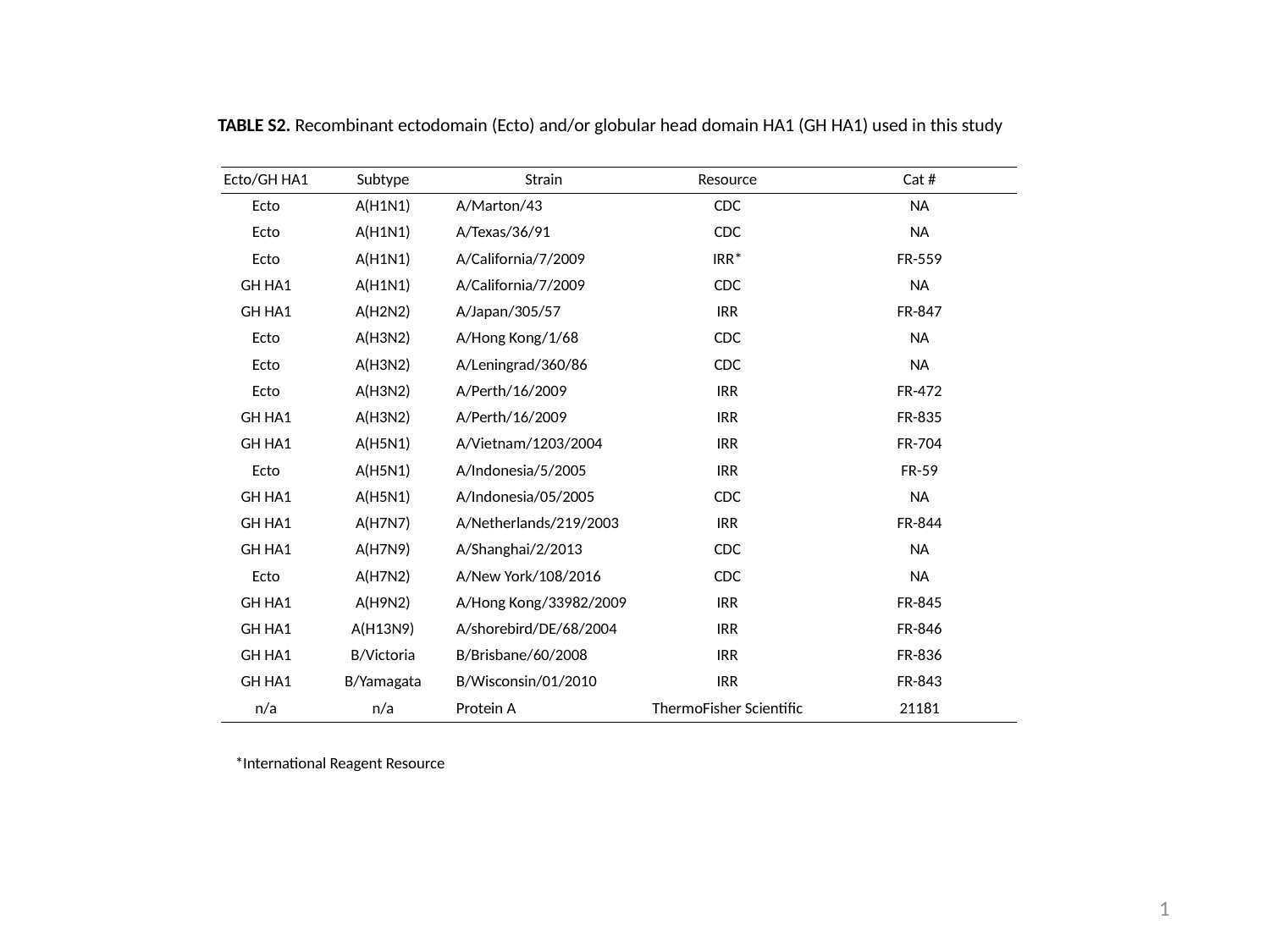

Table S2. Recombinant ectodomain (Ecto) and/or globular head domain HA1 (GH HA1) used in this study
| | | | | |
| --- | --- | --- | --- | --- |
| Ecto/GH HA1 | Subtype | Strain | Resource | Cat # |
| Ecto | A(H1N1) | A/Marton/43 | CDC | NA |
| Ecto | A(H1N1) | A/Texas/36/91 | CDC | NA |
| Ecto | A(H1N1) | A/California/7/2009 | IRR\* | FR-559 |
| GH HA1 | A(H1N1) | A/California/7/2009 | CDC | NA |
| GH HA1 | A(H2N2) | A/Japan/305/57 | IRR | FR-847 |
| Ecto | A(H3N2) | A/Hong Kong/1/68 | CDC | NA |
| Ecto | A(H3N2) | A/Leningrad/360/86 | CDC | NA |
| Ecto | A(H3N2) | A/Perth/16/2009 | IRR | FR-472 |
| GH HA1 | A(H3N2) | A/Perth/16/2009 | IRR | FR-835 |
| GH HA1 | A(H5N1) | A/Vietnam/1203/2004 | IRR | FR-704 |
| Ecto | A(H5N1) | A/Indonesia/5/2005 | IRR | FR-59 |
| GH HA1 | A(H5N1) | A/Indonesia/05/2005 | CDC | NA |
| GH HA1 | A(H7N7) | A/Netherlands/219/2003 | IRR | FR-844 |
| GH HA1 | A(H7N9) | A/Shanghai/2/2013 | CDC | NA |
| Ecto | A(H7N2) | A/New York/108/2016 | CDC | NA |
| GH HA1 | A(H9N2) | A/Hong Kong/33982/2009 | IRR | FR-845 |
| GH HA1 | A(H13N9) | A/shorebird/DE/68/2004 | IRR | FR-846 |
| GH HA1 | B/Victoria | B/Brisbane/60/2008 | IRR | FR-836 |
| GH HA1 | B/Yamagata | B/Wisconsin/01/2010 | IRR | FR-843 |
| n/a | n/a | Protein A | ThermoFisher Scientific | 21181 |
| | | | | |
| \*International Reagent Resource | | | | |
1
