# Supplementary material for: Identification of novel influenza A virus exposures by an improved high‐throughput multiplex MAGPIX platform and serum adsorption
Source: Influenza Other Respir Viruses. 2019 Nov 8;14(2):129–41. doi: 10.1111/irv.12695 (PMC7040970; doi:10.1111/irv.12695)
Supplement: Supplementary file 7 [file IRV-14-129-s007.pptx]

## Slide 1
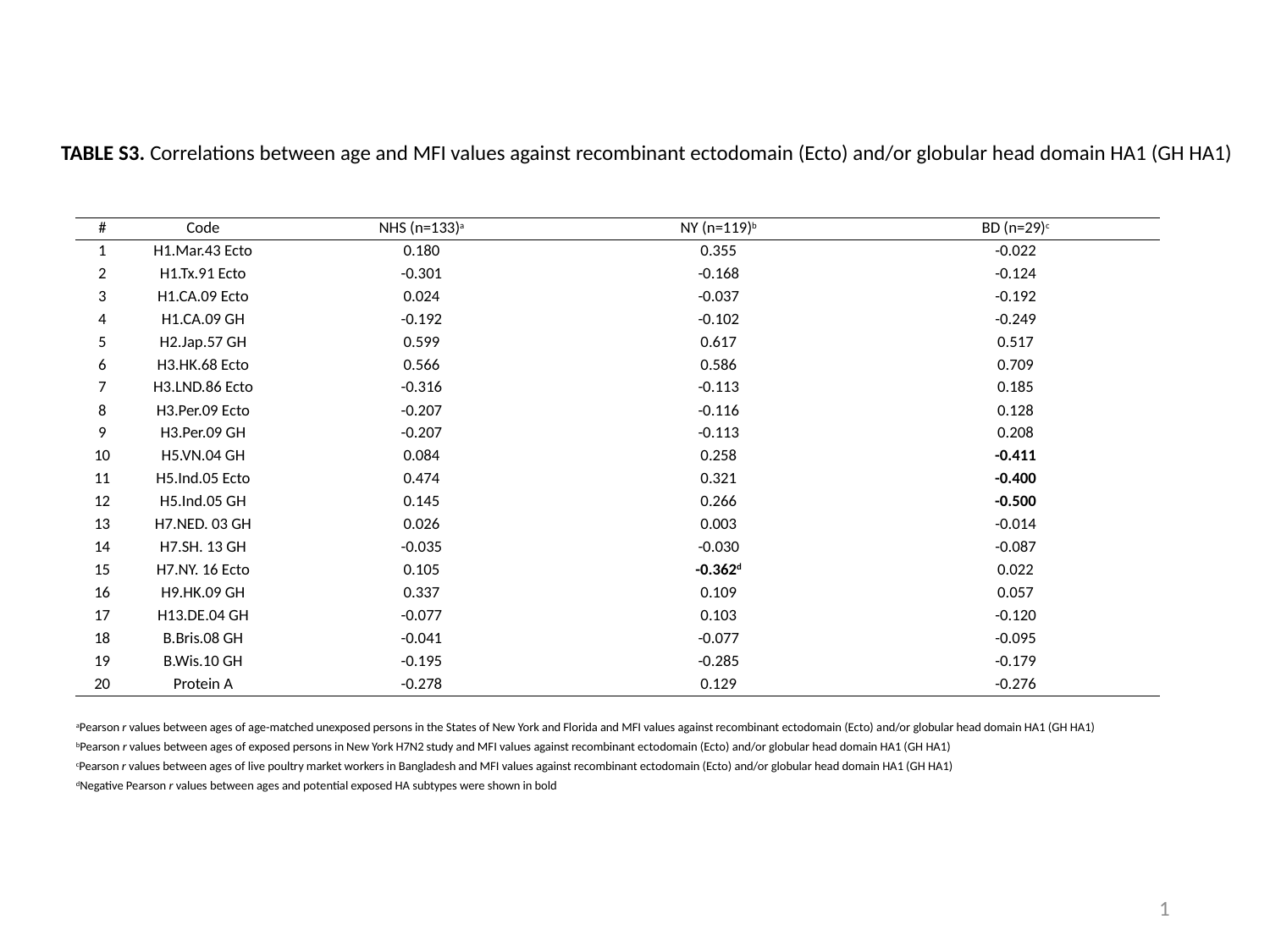

TABLE S3. Correlations between age and MFI values against recombinant ectodomain (Ecto) and/or globular head domain HA1 (GH HA1)
| | | | | | | |
| --- | --- | --- | --- | --- | --- | --- |
| | | | | | | |
| # | Code | NHS (n=133)a | | NY (n=119)b | | BD (n=29)c |
| 1 | H1.Mar.43 Ecto | 0.180 | | 0.355 | | -0.022 |
| 2 | H1.Tx.91 Ecto | -0.301 | | -0.168 | | -0.124 |
| 3 | H1.CA.09 Ecto | 0.024 | | -0.037 | | -0.192 |
| 4 | H1.CA.09 GH | -0.192 | | -0.102 | | -0.249 |
| 5 | H2.Jap.57 GH | 0.599 | | 0.617 | | 0.517 |
| 6 | H3.HK.68 Ecto | 0.566 | | 0.586 | | 0.709 |
| 7 | H3.LND.86 Ecto | -0.316 | | -0.113 | | 0.185 |
| 8 | H3.Per.09 Ecto | -0.207 | | -0.116 | | 0.128 |
| 9 | H3.Per.09 GH | -0.207 | | -0.113 | | 0.208 |
| 10 | H5.VN.04 GH | 0.084 | | 0.258 | | -0.411 |
| 11 | H5.Ind.05 Ecto | 0.474 | | 0.321 | | -0.400 |
| 12 | H5.Ind.05 GH | 0.145 | | 0.266 | | -0.500 |
| 13 | H7.NED. 03 GH | 0.026 | | 0.003 | | -0.014 |
| 14 | H7.SH. 13 GH | -0.035 | | -0.030 | | -0.087 |
| 15 | H7.NY. 16 Ecto | 0.105 | | -0.362d | | 0.022 |
| 16 | H9.HK.09 GH | 0.337 | | 0.109 | | 0.057 |
| 17 | H13.DE.04 GH | -0.077 | | 0.103 | | -0.120 |
| 18 | B.Bris.08 GH | -0.041 | | -0.077 | | -0.095 |
| 19 | B.Wis.10 GH | -0.195 | | -0.285 | | -0.179 |
| 20 | Protein A | -0.278 | | 0.129 | | -0.276 |
| | | | | | | |
| aPearson r values between ages of age-matched unexposed persons in the States of New York and Florida and MFI values against recombinant ectodomain (Ecto) and/or globular head domain HA1 (GH HA1) | | | | | | |
| bPearson r values between ages of exposed persons in New York H7N2 study and MFI values against recombinant ectodomain (Ecto) and/or globular head domain HA1 (GH HA1) | | | | | | |
| cPearson r values between ages of live poultry market workers in Bangladesh and MFI values against recombinant ectodomain (Ecto) and/or globular head domain HA1 (GH HA1) | | | | | | |
| dNegative Pearson r values between ages and potential exposed HA subtypes were shown in bold | | | | | | |
1
